# Machine Learning and Its Application in Skin Cancer

**DOI:** 10.3390/ijerph182413409

**Published:** 2021-12-20

**Authors:** Kinnor Das, Clay J. Cockerell, Anant Patil, Paweł Pietkiewicz, Mario Giulini, Stephan Grabbe, Mohamad Goldust

**Affiliations:** 1 Department of Dermatology Venereology and Leprosy, Silchar Medical College, Silchar 788014, India; daskinnor.das@gmail.com; 2 Departments of Dermatology and Pathology, The University of Texas Southwestern Medical Center, Dallas, TX 75390, USA; ccockerell@dermpath.com; 3 Cockerell Dermatopathology, Dallas, TX 75235, USA; 4 Department of Pharmacology, Dr. DY Patil Medical College, Navi Mumbai 400706, India; anantd1patil@gmail.com; 5 Surgical Oncology and General Surgery Clinic I, Greater Poland Cancer Center, 61-866 Poznan, Poland; 6 Department of Dermatology, University Medical Center Mainz, Langenbeckstraße 1, 55131 Mainz, Germany; mario.giulini@unimedizin-mainz.de (M.G.); stephan.grabbe@unimedizin-mainz.de (S.G.)

**Keywords:** artificial intelligence, machine learning, skin cancer

## Abstract

Artificial intelligence (AI) has wide applications in healthcare, including dermatology. Machine learning (ML) is a subfield of AI involving statistical models and algorithms that can progressively learn from data to predict the characteristics of new samples and perform a desired task. Although it has a significant role in the detection of skin cancer, dermatology skill lags behind radiology in terms of AI acceptance. With continuous spread, use, and emerging technologies, AI is becoming more widely available even to the general population. AI can be of use for the early detection of skin cancer. For example, the use of deep convolutional neural networks can help to develop a system to evaluate images of the skin to diagnose skin cancer. Early detection is key for the effective treatment and better outcomes of skin cancer. Specialists can accurately diagnose the cancer, however, considering their limited numbers, there is a need to develop automated systems that can diagnose the disease efficiently to save lives and reduce health and financial burdens on the patients. ML can be of significant use in this regard. In this article, we discuss the fundamentals of ML and its potential in assisting the diagnosis of skin cancer.

## 1. Introduction

Cancer is one of the major healthcare burdens across the world. Global statistics suggest almost 10.0 million deaths (9.9 million excluding non-melanoma skin cancer) due to cancer in the year 2020. The most commonly diagnosed cancers include breast cancer in females, lung cancer, and prostate cancers. Lung, liver, and stomach cancers are the major contributors of cancer related deaths [1]. Skin cancer, including both malignant melanoma and non-melanoma skin cancer (NMSC), are common cancers in Caucasians and their incidence is on the rise [2]. According to the US Skin Cancer Foundation, skin cancer affects more people in the United States each year than all other cancers combined [3].

Melanoma is the skin cancer with the worst prognosis. If diagnosed early, it can be treated successfully with surgical procedures. However, once there is metastasis, rates of survival are reduced significantly [4]. Diagnosis of melanoma depends on the clinical examination and classic findings on the lesion biopsy. Examples of NMSC include basal cell carcinoma (NMSC) and squamous cell carcinoma. The success of skin cancer depends on early diagnosis and appropriate treatment. Visual inspection may not be sufficient to differentiate benign lesions from malignant tumors. The gold standard procedure is histopathology examination of the skin biopsy. The invasive nature of the procedure, associated pain, and the need for repeated samples in suspected lesions with varied presentations are some of the limitations for skin biopsy. Non-invasive tools can also assist in clinical diagnosis [5]. Expertise, cost, and availability are the challenges for the widespread use of these tools. Several advancements in science and technology have resulted in the availability of different non-invasive imaging methods to detect melanoma [4]. The accuracy of these methods in the diagnosis of melanoma and other skin cancers is still a point of discussion.

Overall, early detection is key for the effective treatment and better outcomes of skin cancers. Specialists can accurately diagnose the cancer, however, considering their limited numbers, there is a need to develop automated systems, which can diagnose the disease efficiently to save lives and reduce health and financial burdens on the patients. Skin tumors can be difficult to recognize from common benign skin lesions, and melanoma has a particularly varied look. AI can aid in the early detection of skin cancer, lowering the burden of morbidity and mortality associated with the disease [6]. In addition to reducing the workload, AI-based systems can also help by improving skin lesion diagnostics [7,8].

Artificial intelligence (AI), a branch of computer science that uses machines and programs to mimic intelligent human behavior via a constellation of technologies, is a key driver of the fourth industrial revolution. Machine learning (ML) is an AI technique involving statistical models and algorithms that can progressively learn from data to predict the characteristics of new samples and perform a desired task. Thus, the complex algorithms are designed to perform the tasks that otherwise would be difficult for human brains to do. Convolutional neural network (CNN) is a type of ML that simulates the processing of biological neurons and is the state-of-the-art network for pattern recognition in medical image analysis. AI is poised to bring transformation in healthcare because of its advantages over traditional analytical techniques. There is rising optimism regarding applications of AI in healthcare, ranging from assistance in medical diagnostics, treatment and administrative support to reduce timelines of new drug development. It may also be of benefit as an adjuvant in clinical decision making [9]. Dermatology, as a visually intensive field, is at the precipice of an AI revolution. The association for the advancement of AI defines it as “the scientific knowledge of the mechanisms underlying mind and intelligent behavior and its implementation in machines” [10]. AI uses computer systems to accomplish tasks that would ordinarily need human intelligence, such as identifying the type of flower or recognizing a person’s voice. To emulate the actions of the human brain, AI uses a variety of technologies and techniques, including robotics, ML, and the internet. AI has the potential to exceed humans, due to its endless processing power and storage capacity [11]. Apple’s Siri, Amazon’s Alexa, and Google Assistant are the most popular instances of AI currently in use by ordinary people [12].

Because skin disease diagnosis is mostly based on visual perception, computer vision algorithms may be able to recognize skin lesions based on their morphology.

By September 2018, the US Food and Drug Administration (FDA) had authorized AI approaches for clinical usage, including devices to detect skin cancer from clinical photos obtained via a smartphone app [13].

The field of AI is growing dynamically, and research in this area is evolving at a rapid speed. The objective of this article is to provide update on usefulness of AI in diagnosis and management of skin cancer. We reviewed the latest research and key discoveries in ML encompassing various subfields of dermatology related cancers. Literature review was performed to screen the articles published in “PubMed” and “Google Scholar” through August 2021. The search words included “Artificial intelligence AND skin cancer” “Machine learning AND skin cancer” and “Deep learning AND skin cancer”. Relevant references of the screened articles were also included for qualitative analysis. Important websites related to skin cancer and related AI resources were also browsed to gather information on the topic.

## 2. Basics of Machine Learning and Deep Learning

According to the technology-oriented approach, AI can be classified into three types, namely artificial narrow intelligence, general artificial intelligence, and artificial superintelligence [14]. Narrow AI is capable of performing a specific task intelligently. The use of narrow AI is the most frequent. General AI is capable of doing any intellectual task like a human being. Super AI may exceed human capabilities and accomplish any piece of work better than humans with cognitive qualities. We currently only have access to weak or narrow AI, such as Apple’s SIRI, which trains a machine to execute a specific task. Reactive machines are the AI systems that do not preserve experiences to perform any activities in the future. They just consider present circumstances and retaliate in the best way. Examples of these include IBM’s Deep Blue system and Google’s AlphaGo. AI machines with limited memory (e.g., self-driving cars) can preserve experiences or data for a finite time period. Theory of mind comprehends human emotions, individuals, and beliefs, as well as the ability to interact socially. This form of AI machine is yet to be created.

ML is a division of AI where computer systems learn from their experiences without having to be explicitly programmed. A supervised, semi-supervised, or unsupervised process can be used. The machine is fed datasets of problems and answers in a supervised configuration. Through trial and error, machines learn to select the correct response. Machines analyze incoming data with no predetermined solution in unsupervised learning. Semi-supervised learning is a method that uses both labelled and unlabeled data [15]. Deep learning is a category of ML that recruits multiple layered deep neural networks, each of which can recognize and learn distinct features particular to the dataset [16]. An artificial neuron network (ANN) is a computational model based on the structure and operations of biological neural networks [17]. The most basic type of ANN is the feedforward neural network. It has three layers, namely input, hidden, and output, where data goes in via the input layer, crosses the hidden layer, and comes out via the output nodes. Multiple hidden levels are possible [18,19]. CNNs is a variant of deep, feed-forward ANN that is most typically used to analyze visual imagery. It is made up of convolutional as well as pooling layers that allow the network to encode picture characteristics [20].

## 3. Skin Cancer and Deep Learning

Codella et al. used the International Skin Imaging Collaboration (ISIC)-2016 dataset to create a conglomeration of deep learning algorithms and compared them against the performance of eight dermatologists to comprehend 100 skin lesions as either benign or malignant. Their conglomeration outmatched dermatologists, with a precision of 76% and specificity of 62%, compared to a precision of 70.5% and specificity of 59% for dermatologists [21]. Haenssle et al. used a large dermoscopic dataset with more than hundred thousand benign lesions and melanoma captures to train a deep learning algorithm called InceptionV4, and compared its performance with 58 dermatologists. The level of diagnosis was divided into two categories. In the first level, only dermoscopy was used, while in the second category, dermoscopy was used in addition to clinical information and patient images. In the first level, dermatologists reported a median sensitivity of 86.6% and specificity of 71.3%. The sensitivity and specificity in level II increased to 88.9% and 75.7%, respectively. The improvement in the specificity was statistically significant (*p* < 0.05). However, the improvement in the sensitivity was statistically non-significant (*p* = 0.19). The deep learning CNN receiver operating characteristics curve showed a significantly higher specificity than for the dermatologists in level I (*p* < 0.01) and level II (*p* < 0.01). In this study, CNN outperformed most dermatologists, suggesting a promising role in the detection of melanoma using dermoscopic images [8]. Another study by Brinker et al. showed similar results. In this study, investigators used a convolutional neural network (ResNet50) to compare the efficacy of 157 dermatologists on hundred dermoscopic images (MClass-D). The dermatologists had an overall sensitivity of 74.1% and a specificity of 60.0%, whereas the deep learning method had a specificity of 69.2% and a sensitivity of 84.2%. In a head-to-head comparison, the performance of CNN was better than 86.6% of dermatologists in the study. The performance was better across subgroups of dermatologists based on experience in the classification of dermoscopv melanoma images [22]. Thus, CNN has significant potential to assist dermatologists in the accurate diagnosis of melanoma. Tschandl et al. used convolutional neural networks such as InceptionV3 and ResNet50 to diagnose non-pigmented skin malignancies using a mixed dataset of 7895 dermoscopic and 5829 close-up lesion photos [23]. The results were compared to those of 95 dermatologists, separated into three groups based on experience. With beginning and intermediate raters, the deep learning algorithms attained an accuracy like humans and outmatched the human groups. The area under the ROC curve of the trained combined CNN was significantly higher than for the dermatologists. It showed correct diagnoses in a higher percentage of cases than for overall dermatologists, but not compared to the experts, i.e., more than 10 years of experience. Maron et al. tested the sensitivity and specificity of a ResNet50 deep learning system for multiclass categorization of skin lesions, along with 112 German dermatologists. The sensitivity and specificity of primary end-point of correct classification of skin lesions for dermatologists was 74.4% and 59.8%, respectively. At a similar level of sensitivity, the specificity of the algorithm was 91.3%. For the secondary end point of correctly classifying a given image into one of the five diagnostic classes, dermatologists had a sensitivity and specificity of 56.5% and 89.2%, respectively. At a similar sensitivity level, the algorithm provided 98.8% specificity. Overall, for the primary end point, dermatologists were significantly outmatched by the deep learning algorithm (*p* < 0.001). The comparison for the secondary end point also showed an outperformance of the algorithm over dermatologists in all categories, except basal cell carcinoma, for which the algorithm had similar performance as that of dermatologists [24]. On a dermoscopic test set of 100 instances, Haenssle et al. weighed up InceptionV4-based deep learning architecture with dermatologists. This study had two levels: level I was a dermoscopic image, and level II had a clinical close-up image, a dermoscopic image, and clinical information. The deep learning system had a sensitivity of 95% and specificity of 76.7%; however, the dermatologists in level I had a mean sensitivity of 89% and specificity of 80.7%, respectively. The dermatologists’ mean sensitivity reached 94.1% with extra information in level II, while their mean specificity remained same [25]. Tschandl et al. conducted an open, web-based study to diagnose the dermatoscoping images. The investigators juxtaposed the average potential of the AI algorithms (139 in total) and 511 human readers on an experimental 1511 set of photos in the ISIC 2018 competition. The diagnoses (seven predefined categories) provided by the humans were compared with those from the algorithms prepared from machine learning. The differences in the percentage of correct diagnoses were compared. Out of the human participants, 55.4%, 23.1%, and 16.2% were board-certified dermatologists, residents of dermatology, and general practitioners, respectively. The results showed a mean of 2.01 for more correct diagnoses by the algorithms than the humans. The difference was statistically significant (*p* < 0.0001). As a result, the AI algorithms were able to make more accurate diagnoses than the human readers [26].

## 4. Algorithms for Machine Learning in Skin Cancer

Because of the high prevalence of skin malignancies, an increasing number of people require prompt diagnosis and ongoing monitoring. This places a huge strain on specialist medical services, which may be allayed by better patient self-surveillance techniques as well as the use of decision support systems for less experienced physicians. Machine diagnosis is not subjective, and is not impacted by external factors. However, human diagnosis is associated with subjective variations and may be impacted by some external factors. If implemented with the necessary regulations, the use of AI for the detection and progression of skin cancer may result in fewer biopsies. Following a training intervention, patients with skin cancer and their guardians can perform self-skin examination (SSE). This also boosts teledermoscopy, leading to fewer medical consultations.

The inclusion of AI in smartphone applications can teach people to perform skin examination and forward the information to the physician. Each form of skin lesion is assigned a class, such as “benign” and “malignant”, or “naevi” and “melanoma”, in oder to construct a new ML skin cancer algorithm. Deep learning algorithms are taught on a large number of photos in each class before being evaluated on a new image. There are three basic parts to the procedure. In the first stage, the algorithm is fed digitized macroscopic or dermoscopic images labelled with the “ground truth” in the first stage (in this case, the ground truth is the lesion diagnosis, which is determined by an experienced dermatologist or by histological study). In stage 2, convolutional layers extract the feature map from the images. A feature map is a visual representation of the data, which has several degrees of abstraction. Low-level features such as edges, corners, and forms are extracted by the first convolutional layers. To recognize the type of skin lesion, later convolutional layers extract high-level data. The machine learning classifier uses the feature maps in stage 3 to recognize distinct kinds of skin lesion patterns. A fresh image can now be classified using the deep learning method.

## 5. Skin Cancer Datasets

Particularly in dermatology, clinical and dermatoscopic images are often generated to track changes in skin conditions. New applications will make the gigantic amounts of data that already exist and will be created in the future, e.g., in hospitals, accessible to algorithms and lead to an improvement of CNNs. There are already data sets accessible for research. ISIC archive gallery contains numerous of clinical and dermoscopic skin lesion datasets, including the ISIC Challenges datasets, HAM10000, and BCN20000 [27,28,29]. Interactive Atlas of Dermoscopy has 1000 clinical examples including 270 melanomas and 49 seborrheic keratoses. Each case has a minimum of two images—dermoscopic and close-up. Its price is €250 and is available for research purposes [30]. Dermofit Image Library has 1300 high-resolution photographs of skin lesions divided into 10 categories. A licensing agreement is required, with a one-time license charge with the availability of academic license [31]. PH2 Dataset contains 200 dermoscopic images, including 40 melanoma and 160 nevi cases. It is free for downloading after the completion of an online registration form [32]. MED-NODE Dataset contains 170 clinical photos, including 70 melanoma and 100 nevi cases. This dataset can be downloaded without any cost for research purposes [33]. Asan Dataset contains 17,125 clinical photos of 12 different forms of skin illnesses that affect Asians. It is available to download for research purposes [34,35]. The Hallym Dataset has 125 clinical photos of BCC cases (34Han JID). SD-198 dataset contains 6584 clinical photos of 198 skin illnesses. The 25 SD-260 dataset is more balanced than SD-198 dataset, since it manages the class size distribution while preserving 10–60 photos for each category. There are 20,600 photos in all, representing 260 skin illnesses [36]. Dermnet NZ is the source of one of the most comprehensive and diverse collections of clinical, dermoscopic, and histology photographs. Additional high-resolution pictures are available for purchase [37]. Derm7pt contains 1011 dermoscopic images including 252 melanoma and 759 nevi cases based on a seven-point checklist [38]. The Cancer Genome Atlas has 2871 pathological skin lesion slides, making it one of the largest collections of its kind. It is openly available for usage by the research community [39].

## 6. Deep Learning and Clinical Images

Clinical photos of various skin lesions are routinely captured using cell phone cameras for remote assessment and assimilation into patient medical records. On the SD-198 dataset, Yang et al. achieved clinically observed skin lesion identification utilizing the well-known ABCD rule. They compared the performance of deep learning methods with dermatologist outputs. It received 57.62% accuracy compared to the 53.35% accuracy for the best performing deep learning system (ResNet). Only senior experienced clinicians had an average accuracy of 83.29% when compared to the rest of the clinicians [40]. Han et al. used a MED-NODE dataset and atlas site images for training a deep learning architecture (ResNet-152) to differentiate clinical photos of 12 skin illnesses, and then examined it on an Asan testing set and an Edinburgh Dataset (Dermofit). Upon taking 480 random photos merged from the Asan test dataset (260 images) and the Edinburgh dataset (220 images), the algorithm’s performance was equivalent to a team of 16 dermatologists, but the AI system outclassed dermatologists while diagnosing basal cell carcinoma (BCC) [34]. Fujisawa et al. trained deep CNN with 4867 clinical images from 14 skin diseases from 1842 patients with different diagnoses, including malignant and benign diseases, to evaluate a deep learning algorithm. The results of the algorithm were compared with those of the dermatologists. The deep learning algorithm produced a diagnostic accuracy, sensitivity, and specificity of 76.5%, 96.3%, and 89.5%, respectively. The accuracy of classifying images into the benign or malignant category by the dermatologists board certified dermatologists (*n* = 13), dermatology trainees (*n* = 9), and deep CNN was 85.3%, 74.4%, and 92.4%, respectively. The performance by the board-certified dermatologists was significantly better than for the trainees. However, the accuracy of deep CNN was higher than for both human raters [41]. In a test case of 100 clinical skin lesion photographs (MClass-ND), Brinker et al. evaluated 145 dermatologist performances and a deep learning approach (ResNet50) for the 80 nevi cases and 20 histopathologically proven melanoma cases. The dermatologists had sensitivity of 89.4% and a specificity of 64.44%, while a deep learning technique at the same sensitivity had a mean specificity score of 68.2% [42]. Overall, the CNN performance was on par with dermatologists in terms of the classification of clinical images. Variance with CNN was smaller, suggesting a greater robustness of AI than humans for the classification of images. Only 19 (13.1%) dermatologists had a higher sensitivity than the CNN. Out of these 19 dermatologists, 16 (84.2%) achieved a sensitivity of more than 95%.

## 7. Deep Learning and Histopathology Images

Dermatopathologists confirm the diagnosis of skin cancer through histopathologic examination of a tissue biopsy under a microscope. One of the important challenges in the confirmatory diagnosis of skin cancer is the high rates of discordance between different pathologists. In the case of the diagnosis of melanoma, there can be discordance in classifying whether it is a benign or malignant lesion. With whole-slide imaging, deep learning methodologies have been successful for digital pathology. These methods are used to classify biopsy tissue specimens in order to diagnose malignancies. Different investigators have performed studies to compare the performance of an expert versus that of AI system. Heckler et al. [43] compared pathologists’ performance for identifying melanoma and nevi using a deep learning approach. The study included 695 lesions classified by an expert, of which 595 were used for training the CNN. The remaining 100 were used to test the results of CNN with those of the 11 experts. In this study, the investigators digitalized the entire slides. The image sections with magnification were randomly cropped. The sensitivity, specificity, and accuracy of the CNN was compared with that of the pathologists. In a recently published study, Brinker et al. [44] reported comparative results of the ability of CNN to differentiate melanomas from nevi using hematoxylin−eosine stained whole slide images (WSI). In this study involving whole slide images of 50 melanomas and 50 nevi, the performance of CNN was on par with the experts. Jiang et al. [45] came up with a deep learning method for diagnosing BCC using smartphone-captured histopathology images. They found that the algorithm’s performance on smartphone-captured images and WSI was comparable, accompanied by an AUC of 0.95. For an in-depth analysis of the difficult cases, they used a deep segmentation network, which resulted in a score of 0.987 (AUC), 0.97 (sensitivity), and 0.94 (specificity). The work of Jiang and colleagues suggests the usefulness of deep learning methods for the diagnosis of BCC, with a high sensitivity and specificity.

On 1417 images from 308 regions of interest (ROI) of skin histopathology images, Cruz-Roa et al. [46] employed deep learning architecture to identify between BCC and normal tissue patterns. They compared deep learning to classical ML using feature descriptors such as the bag of features, canonical wavelet transform, and Haar-based wavelet transform. The deep learning architecture outperformed previous approaches with an F-Measure of 89.4% and a balanced accuracy of 91.4%. From 2008 to 2018, Xie et al. [47] published a humongous dataset of 2241 histopathology pictures from 1321 individuals. They tested the categorization of melanoma and nevi on various magnification scales by two deep learning architectures, viz. VGG19 and ResNet50, by making use of the 9.95 million patches created on 2241 histopath images. With a mean F1 (0.89), specificity (0.94), sensitivity (0.92), and AUC (0.94), they were able to identify melanoma from nevi with a good accuracy (0.98) [47].

It should be noted that different results from different studies suggest that the amount of data presented to the AI system, the methodology used for the study, and the complexity of disease may affect the level of difficulty for a given task and thus the performance of both AI algorithms and human observers.

Overall, it seems that CNNs can be of valuable assistance to humans for the diagnosis of skin cancers such as melanoma. Similarly, the diagnosis of BCC needs intensive work because of the need to examine a large number of images. Deep learning methods can be of use to assist the diagnosis of BCC. WSIs and microscopic ocular images with use of smartphone cameras can be useful for developing neural network models for the diagnosis of BCC. A reduced time for the diagnosis and cost benefit are some of the advantages of CNN in the diagnosis of skin cancers.

## 8. AI Acceptance by Patients and Clinicians

In a recent study, Jutzi and colleagues [6] conducted a survey-based study to understand the views of the patients in Germany towards AI in the diagnosis of melanoma. In this study involving 298 participants, 154 (51.7%) had a diagnosis of melanoma. Interestingly, most of the respondents (94%) supported the use of AI in healthcare. This is a very encouraging finding, as the acceptance of the patient plays an important role in the success of any healthcare related decisions. In line with the point discussed before, sharing data is necessary for better results from the AI. The results of this survey suggested that 88% of participants were aggregable to provide their own health related records for the development of AI-based applications. Another important finding of this study was that patients with a history of melanoma were more inclined to use AI applications for early diagnosis. Another qualitative study involving 48 patients reported a receptive approach towards the use of AI for the screening of skin cancer, provided it was used in a way that ensured the integrity of the doctor−patient relationship [48]. A study by Oh et al. [49] reported good familiarity of AI by only 5.9% of physicians out of 669 who completed the questionnaire-based study. Interestingly, 83.4% considered AI useful in healthcare. Similarly, a number of participants agreed that disease diagnosis is the most promising area for the use of AI in healthcare. According to 43.9% of participants, AI is superior in the diagnosis of disease compared with doctors. We did not find any study specifically evaluating the acceptance of ML in skin cancer diagnosis by dermatologists. We feel that if increased accuracy and early detection is possible with ML, it may be well accepted by the clinicians. AI will not replace clinicians, but rather assist them in better evaluation and hence management of patients. Although the literature suggests the usefulness of ML in skin cancer diagnosis, its applications will largely depend on acceptance by the clinicians.

## 9. Future Prospects

AI has a wide scope in healthcare settings, for both diagnosis and therapeutic purposes. One of the major challenges for AI is the need for training the machine learning approach with the continuous feeding of data. Clinicians and patients should be aggregable to continuously provide images for better results with AI applications. Anonymity and privacy should be taken seriously when feeding the data into AI systems. Harmonization of the regulatory norms across the globe will be the key to the widespread use of AI systems in healthcare. Larger studies among dermatologists are needed to provide more insights about their perceptions and acceptance of ML in the diagnosis of skin cancers.

## 10. Conclusions

In the discipline of dermatology, AI is quickly gaining traction. It has the potential to transform patient care, especially in terms of enhancing the sensitivity and accuracy of screening for skin lesions, including cancer. However, AI research requires clinical and photographic data of all skin types, which can only be obtained through improved worldwide skin imaging collaboration. The sensitivities, specificities, and performance need to be documented in prospective studies and real-life settings. Dermatologists should not see AI as a threat to their expertise; rather, in the next years, it can be used as a supplement to clinical practice. Practicing dermatologists will be able to provide better skin care if they have a better understanding of AI ideas. The challenges in the implementation of AI for the diagnosis of skin cancer include protection of patient data, availability of large datasets, and training the AI algorithms to reduce the rates of errors and increase accuracy of diagnosis. The limited available data from survey-based research suggests a positive attitude of patients towards the use of AI for the diagnosis of melanoma. Further research is necessary in these directions.

## Data Availability

Not applicable.

## References

[1] Ferlay J., Colombet M., Soerjomataram I., Parkin D.M., Pineros M., Znaor A., Bray F. (2021). Cancer statistics for the year 2020: An overview. Int. J. Cancer.

[2] Apalla Z., Nashan D., Weller R.B., Castellsaque X. (2017). Skin cancer: Epidemiology, disease burden, pathophysiology, diagnosis and therapeutic approaches. Dermatol. Ther..

[3] Skin Cancer Facts & Statistics [Internet]. The Skin Cancer Foundation. https://www.skincancer.org/skin-cancer-information/skin-cancer-facts/.

[4] Davis L.E., Shalin S.C., Tackett A.J. (2019). Current state of melanoma diagnosis and treatment. Cancer Biol. Ther..

[5] Malvehy J., Pellacani G. (2017). Dermoscopy, confocal microscopy and other non-invasive tools for the diagnosis of non-melanoma skin cancers and other skin conditions. Acta Derm. Venereol..

[6] Jutzi T.B., Krieghoff-Henning E.I., Holland-Letz T., Utikal J.S., Hauschild A., Schadendorf D., Sondermann W., Fröhling S., Hekler A., Schmitt M. (2020). Artificial Intelligence in Skin Cancer Diagnostics: The Patients’ Perspective. Front. Med..

[7] Sengupta S., Mittal N., Modi M. (2020). Improved skin lesions detection using color space and artificial intelligence techniques. J. Dermatolog. Treat..

[8] Haenssle H.A., Fink C., Schneiderbauer R., Toberer F., Buhl T., Blum A., Kalloo A., Hassen A.B.H., Thomas L., Enk A. (2018). Man against machine: Diagnostic performance of a deep learning convolutional neural network for dermoscopic melanoma recognition in comparison to 58 dermatologists. Ann. Oncol..

[9] Bohr A., Memarzadeh K. (2020). The rise of artificial intelligence in healthcare applications. Artif. Intell. Healthc..

[10] Hernández-Orallo J., Martínez-Plumed F., Schmid U., Siebers M., Dowe D.L. (2016). Computer models solving intelligence test problems: Progress and implications. Artif. Intell..

[11] Goyal M. Artificial Intelligence in Dermatology|DermNet NZ [Internet]. https://dermnetnz.org/topics/artificial-intelligence/.

[12] Utermohlen K. (2018). Artificial Intelligence (AI) and the 13 Stats that Prove the Future is Here [Internet]. https://medium.com/@karl.utermohlen/artificial-intelligence-ai-and-the-13-stats-that-prove-the-future-is-here-38e627bb788c.

[13] Chan S., Reddy V., Myers B., Thibodeaux Q., Brownstone N., Liao W. (2020). Machine learning in dermatology: Current applications, opportunities, and limitations. Dermatol. Ther..

[14] Hassani H., Silva E.S., Unger S., TajMazinani M., Mac Feely S. (2020). Artificial intelligence (AI) or intelligence augmentation (IA): What is the future?. AI.

[15] Du-Harpur X., Watt F.M., Luscombe N.M., Lynch M.D. (2020). What is AI? Applications of artificial intelligence to dermatology. Br. J. Dermatol..

[16] Bini S.A. (2018). Artificial Intelligence, Machine Learning, Deep Learning, and Cognitive Computing: What Do These Terms Mean and How Will They Impact Health Care?. J. Arthroplast..

[17] Lin J.W. (2017). Artificial neural network related to biological neuron network: A review. Adv. Stud. Med. Sci..

[18] Bebis G., Georgiopoulos M. (1994). Feed-forward neural networks. IEEE Potentials.

[19] Schmidhuber J. (2015). Deep learning in neural networks: An overview. Neural. Netw..

[20] Gu J., Wang Z., Kuen J., Ma L., Shahroudy A., Shuai B., Liu T., Wang X., Wang G., Cai J. (2018). Recent advances in convolutional neural networks. Pattern Recognit..

[21] Codella N.C.F., Nguyen Q.B., Pankanti S., Gutman D.A., Helba B., Halpern A.C., Smith J.R. (2017). Deep learning ensembles for melanoma recognition in dermoscopy images. IBM J. Res. Dev..

[22] Brinker T.J., Hekler A., Enk A.H., Klode J., Hauschild A., Berking C., Schilling B., Haferkamp S., Schadendorf D., Holland-Letz T. (2019). Deep learning outperformed 136 of 157 dermatologists in a head-to-head dermoscopic melanoma image classification task. Eur. J. Cancer.

[23] Tschandl P., Rosendahl C., Akay B.N., Argenziano G., Blum A., Braun R.P., Cabo H., Gourhant J.Y., Kreusch J., Lallas A. (2019). Expert-level diagnosis of nonpigmented skin cancer by combined convolutional neural networks. JAMA Dermatol..

[24] Maron R.C., Weichenthal M., Utikal J.S., Hekler A., Berking C., Hauschild A., Enk A.H., Haferkamp S., Klode J., Schadendorf D. (2019). Systematic outperformance of 112 dermatologists in multiclass skin cancer image classification by convolutional neural networks. Eur. J. Cancer.

[25] Haenssle H.A., Fink C., Toberer F., Winkler J., Stolz W., Deinlein T., Hofmann-Wellenhof R., Lallas A., Emmert S., Buhl T. (2020). Man against machine reloaded: Performance of a market-approved convolutional neural network in classifying a broad spectrum of skin lesions in comparison with 96 dermatologists working under less artificial conditions. Ann. Oncol..

[26] Tschandl P., Codella N., Akay B.N., Argenziano G., Braun R.P., Cabo H., Gutman D., Halpern A., Helba B., Hofmann-Wellenhof R. (2019). Comparison of the accuracy of human readers versus machine-learning algorithms for pigmented skin lesion classification: An open, web-based, international, diagnostic study. Lancet Oncol..

[27] Codella N.C.F., Gutman D., Celebi M.E., Helba B., Marchetti M.A., Dusza S.W., Kalloo A., Liopyris K., Mishra N., Kittler H. Skin lesion analysis toward melanoma detection: A challenge at the 2017 International symposium on biomedical imaging (ISBI), hosted by the international skin imaging collaboration (ISIC). Proceedings of the 2018 IEEE 15th International Symposium on Biomedical Imaging (ISBI 2018).

[28] Tschandl P., Rosendahl C., Kittler H. (2018). The HAM10000 dataset, a large collection of multi-source dermatoscopic images of common pigmented skin lesions. Sci. Data.

[29] Combalia M., Codella N.C.F., Rotemberg V., Helba B., Vilaplana V., Reiter O., Carrera C., Barreiro A., Halpern A.C., Puig S. (2019). BCN20000: Dermoscopic Lesions in the Wild. http://arxiv.org/abs/1908.02288.

[30] Argenziano G., Soyer H.P., De Giorgio V., Piccolo D., Carli P., Delfino M., Ferrari A., Hofmann-Wellenhof R., Massi D., Mazzocchetti G. (2000). Interactive Atlas of Dermoscopy.

[31] DERMOFIT [Internet]. https://homepages.inf.ed.ac.uk/rbf/DERMOFIT/.

[32] PH2-A Dermoscopic Image Database for Research and Benchmarking [Internet]. https://ieeexplore.ieee.org/abstract/document/6610779/.

[33] Giotis I., Molders N., Land S., Biehl M., Jonkman M.F., Petkov N. (2015). MED-NODE: A computer-assisted melanoma diagnosis system using non-dermoscopic images. Expert Syst. Appl..

[34] Han S.S., Kim M.S., Lim W., Park G.H., Park I., Chang S.E. (2018). Classification of the clinical images for benign and malignant cutaneous tumors using a deep learning algorithm. J. Invest. Dermatol..

[35] Han S.S., Park G.H., Lim W., Kim M.S., Na J.I., Park I., Chang S.E. (2018). Deep neural networks show an equivalent and often superior performance to dermatologists in onychomycosis diagnosis: Automatic construction of onychomycosis datasets by region-based convolutional deep neural network. PLoS ONE.

[36] Yang J., Sun X., Liang J., Rosin P.L. Clinical skin lesion diagnosis using representations inspired by dermatologist criteria. Proceedings of the IEEE Conference on Computer Vision and Pattern Recognition.

[37] DermNet NZ–All about the Skin|DermNet NZ [Internet]. https://www.dermnetnz.org/.

[38] Kawahara J., Daneshvar S., Argenziano G., Hamarneh G. (2019). Seven-point checklist and skin lesion classification using multitask multimodal neural nets. IEEE J. Biomed. Health Inform..

[39] Goyal M., Knackstedt T., Yan S., Hassanpour S. (2020). Artificial intelligence-based image classification methods for diagnosis of skin cancer: Challenges and opportunities. Comput. Biol. Med..

[40] Yang J., Wu X., Liang J., Sun X., Cheng M.M., Rosin P.L., Wang L. (2020). Self-Paced Balance Learning for Clinical Skin Disease Recognition. IEEE Trans. Neural Netw. Learn. Syst..

[41] Fujisawa Y., Otomo Y., Ogata Y., Nakamura Y., Fujita R., Ishitsuka Y., Watanabe R., Okiyama N., Ohara K., Fujimoto M. (2019). Deep-learning-based, computer-aided classifier developed with a small dataset of clinical images surpasses board-certified dermatologists in skin tumour diagnosis. Br. J. Dermatol..

[42] Brinker T.J., Hekler A., Enk A.H., Klode J., Hauschild A., Berking C., Schilling B., Haferkamp S., Schadendorf D., Fröhling S. (2019). A convolutional neural network trained with dermoscopic images performed on par with 145 dermatologists in a clinical melanoma image classification task. Eur. J. Cancer.

[43] Hekler A., Utikal U.S., Enk A.H., Solass W., Schmitt M., Klode J., Schadendorf D., Sondermann W., Franklin C., Bestvater F. (2019). Deep learning outperformed 11 pathologists in the classification of histopathological melanoma images. Eur. J. Cancer.

[44] Brinker T.J., Schmitt M., Krieghoff-Henning E.I., Barnhill R., Beltraminelli H., Braun S.A., Carr R., Fernandez-Figueras M.T., Ferrara G., Fraitag S. (2021). Diagnostic performance of artificial intelligence for histologic melanoma recognition compared to 18 international expert pathologists. J. Am. Acad. Dermatol..

[45] Jiang Y.Q., Xiong J.H., Li H.Y., Yang X.H., Yu W.T., Gao M., Zhao X., Ma Y.P., Zhang W., Guan Y.F. (2020). Recognizing basal cell carcinoma on smartphone-captured digital histopathology images with a deep neural network. Br. J. Dermatol..

[46] Cruz-Roa A.A., Ovalle J.E.A., Madabhushi A., Osorio F.A.G. (2013). A deep learning architecture for image representation, visual interpretability and automated basal-cell carcinoma cancer detection. International Conference on Medical Image Computing and Computer-Assisted Intervention.

[47] Xie P., Zuo K., Zhang Y., Li F., Yin M., Lu K. (2019). Interpretable Classification from Skin Cancer Histology Slides Using Deep Learning: A Retrospective Multicenter Study. http://arxiv.org/abs/1904.06156.

[48] Nelson C.A., Perez-Chada L.M., Creadore A., Li S.J., Lo K., Manjaly P. (2020). Patient perspectives on the use of artificial intelligence for skin cancer screening: A qualitative study. JAMA Dermatol..

[49] Oh S., Kim J.H., Choi S.-W., Lee H.J., Hong J., Kwon S.H. (2019). Physician confidence in artificial intelligence: An online mobile survey. J. Med. Internet Res..

